# The Electro-Oxidation of Ethylene Glycol on Platinum over a Wide pH Range: Oscillations and Temperature Effects

**DOI:** 10.1371/journal.pone.0075086

**Published:** 2013-09-18

**Authors:** Elton Sitta, Raphael Nagao, Hamilton Varela

**Affiliations:** Institute of Chemistry of Sao Carlos, University of Sao Paulo, Sao Carlos, Sao Paulo, Brazil; Northeastern University, United States of America

## Abstract

We report a comprehensive study of the electro-oxidation of ethylene glycol (EG) on platinum with emphasis on the effects exerted by the electrolyte pH, the EG concentration, and temperature, under both regular and oscillatory conditions. We extracted and discussed parameters such as voltammetric activity, reaction orders (with respect to [EG]), oscillation’s amplitude, frequency and waveform, and the evolution of the mean electrode potential at six pH values from 0 to 14. In addition, we obtained the apparent activation energies under several different conditions. Overall, we observed that increasing the electrolyte pH results in a discontinuous transition in most properties studied under both voltammetric and oscillatory regimes. As a relevant result in this direction, we found that the increase in the reaction order with pH is mediated by a minimum (~ 0) at pH = 12. Furthermore, the solution pH strongly affects all features investigated, c.f. the considerable increase in the oscillatory frequency and the decrease in the, oscillatory, activation energy as the pH increase. We suggest that adsorbed CO is probably the main surface-blocking species at low pH, and its absence at high pH is likely to be the main reason behind the differences observed. The size of the parameter region investigated and the amount of comparable parameters and properties presented in this study, as well as the discussion that followed illustrate the strategy of combining investigations under conventional and oscillatory regimes of electrocatalytic systems.

## Introduction

The electrocatalysis of small organic molecules is an active field in electrochemical science due to the possible application of these molecules in low temperature fuel cells [1,2]. Despite the simple structure, the oxidation mechanism of these molecules involves several intermediates that poison the surface and cause the overall kinetic to become sluggish [2]. The competition for free surface sites coupled with the nonlinear dependence between the surface population of these poisoning species and the electrode potential induces the occurrence of chemical instabilities in these systems [3,4,5]. Examples of this phenomenon have been reported during the electro-oxidation of formic acid [6,7,8,9,10,11,12], formaldehyde [11,13,14,15], methanol [14,16,17,18], ethanol [19], ethylene glycol [20,21] and glycerol [22] in both acid and alkaline media (reference [21] provides a deeper description). Interestingly, the electro-oxidation under oscillatory regime can reveal unique features and even contribute to elucidate some mechanistic aspects, as for instance: the temperature (over)compensation phenomenon during the electro-oxidation of formic acid [8] [6]; the possibility of decoupling the parallel pathways [18,23] and, the direct estimation of kinetic parameter [24,25,26].

We have recently reported oscillations during the electro-oxidation of ethylene glycol in alkaline media [20,21,25] showing oscillations with frequencies, at least, ten times higher, ~ 20 Hz, than those observed in other similar systems, with C1-C3 molecules. Previous experiments in acid media also indicated frequencies that lie on ordinary values [21]. In this paper we present a comprehensive study of the effects exerted by the electrolyte pH and temperature on the electro-oxidation of ethylene glycol on platinum. An important aspect that emerges from this work is the observed correspondence between the two sets of data under regular and oscillatory regimes. The amount of comparable parameters and properties presented in this study and the following discussion illustrate the strategy of combining studies under regular and oscillatory conditions for the investigation of electrocatalytic systems.

## Experimental

The electrolyte consists of an aqueous K_2_SO_4_ solution with ionic strength (I_m_) around 1 prepared with different amounts of KOH (Sigma-Aldrich 99.99%) and H_2_SO_4_ (Mallinkrodt 98%). The solution pH was adjusted by dosing the ratio between KOH and H_2_SO_4_. Table 1 shows the measured pH and calculated ionic strength of each solution. Except for slight variations, the values described along the text refer to nominal values. In the range between 2 and 12 the amounts of acid and base were estimated to yield neutral solutions, and then the desired pH was reached by addition of small portions of acid or base. Due to the high salt concentration, this adjustment does not change appreciably the ionic strength. The region between 4 < pH < 10 was avoided because of the abrupt pH changes normally observed in non-buffered solution.

**Table 1:** Measured pH values and calculated ionic strength (I_m_) for the supporting electrolytes.

**Nominal pH**	**Measured pH**	**I_m_**
**0**	0.0	1.02
**2**	2.2	0.80
**4**	3.9	0.99
**10**	9.8	0.99
**12**	12.1	1.00
**14**	14.0	1.00

A platinum band with real area of 0.27 cm^2^, as measured by the H_upd_ region, was used as working electrode, and a high area platinum foil served as counter electrode. For pH values lower than 2 or higher than 12, the reversible hydrogen electrode (RHE) was used as reference, remaining experiments were carried out with a saturated Ag/AgCl/Cl^-^ electrode separated to the cell’s main-compartment by a Luggin capillary. All potential are showed in RHE scale.

The temperature was controlled by a thermostatic bath (Micro-Quimica, model MQBTC99-20) with precision of 0.1 °C. Prior to all experiments the solution was purged with Ar (White Martins 4.8 N) and during the experiments, an Ar atmosphere was kept. The electrode pre-treatment is described elsewhere [21].

## Results and Discussion

### 1 The general effect of pH and [EG]

**Figure 1:**
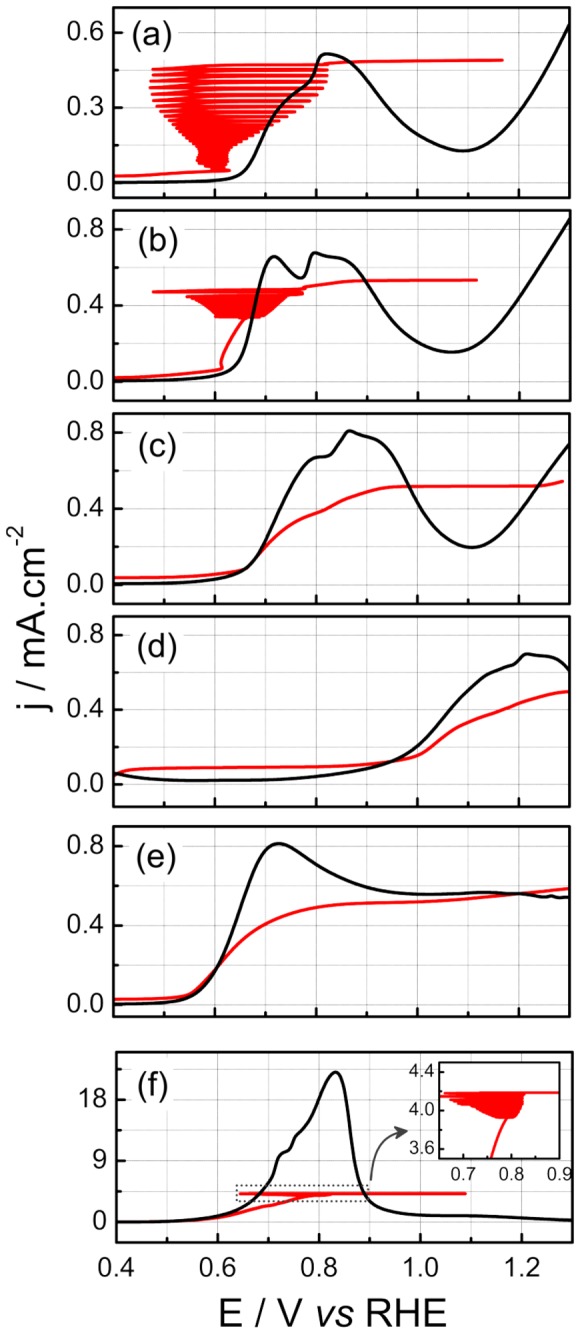
Linear sweep of potential (black) and current (red). dE/dt = 0.01 V.s^-1^, dj/dt = 4 µA s^-1^ cm^-2^, [EG] = 0.5 M, T = 20 ^0^C. (a) to (f) represent the pH of 0, 2, 4, 10, 12 and 14 respectively.

Figure 1 shows the linear potential (black) and current (red) sweeps for the electro-oxidation of 0.5 M of EG at six pH values. Under potential control no oscillations are observed, probably, due to the low uncompensated resistance, i.e. the solution resistance. In all pH studied it is observed more than one oxidation peak, that can appear as a double peak, as in pH = 2; as shoulders; or as a large region after a well-defined peak, c.f. pH = 12. The relative positions of such features in the j/E curves are strongly affected by the electrolyte pH. Roughly speaking, the main oxidation processes occur between 0.6 and 1.0 V, but at pH = 10 it is shifted to higher potentials. The maximum current densities increase with the pH, reaching between 0.5 and 0.8 mA.cm^-2^, at pH between 0 and 12, and 22 mA.cm^-2^ at pH = 14.

The electro-oxidation of CO single layers (CO stripping), in phosphate buffers [27] shows that the onset potential is shifted to lower values as the pH is increased to 11. This trend is apparently not followed by more complicated molecules, such as ethanol [28] in which the main peak keeps around on 0.8 V, in line with EG results, and the current increase from 1.2 to 15 mA.cm^-2^ (at 0.05 Vs^-1^) in the pH range from 1.7 to 12. Systematic studies at different solution pH are non-trivial due to the influence of anion adsorption. It is necessary to add salts to the solution to maintain the ionic strength and also to avoid pH changes (buffers). As well described on literature [29,30] the presence of different anions, certainly plays a role on the overall oxidation process.

Schnaidt et al. [31] studied the electro-oxidation of 0.1 M of EG on platinum by means of *in situ* InfraRed spectroscopy (FTIRS) and Differential Electrochemical Mass Spectrometry (DEMS). Experiments were carried out in 0.5 M H_2_SO_4_ solutions, and they found, at low potentials, mainly adsorbed carbon monoxide and glycolaldehyde as oxidation products. As the potential increases, the oxidation of CO to CO_2_ in the region of the pre-peak (0.75 V) and the production of glycolic acid are found. Around the main oxidation peak, the production of glycolic acid prevails.

In alkaline media, the electro-oxidation of EG on platinum results in carbonate, oxalate, glycolate and glyoxylate, as evidenced by *in situ* FTIRS [32,33,34]. The product distribution strongly depends on the EG concentration and the occurrence of more oxidized products, including the ones with only one carbon atom, prevails at low EG concentrations. In addition to the EG concentration, the alkali cation of the supporting electrolyte also plays an important role on the product distribution [34], and this effect has been rationalized in terms of the non-covalent interaction between cations and adsorbed oxygenated species [35]. We are not aware of previous studies dealing with the product distribution along the electro-oxidation of EG on platinum in the pH range between 2 and 12.

**Figure 2:**
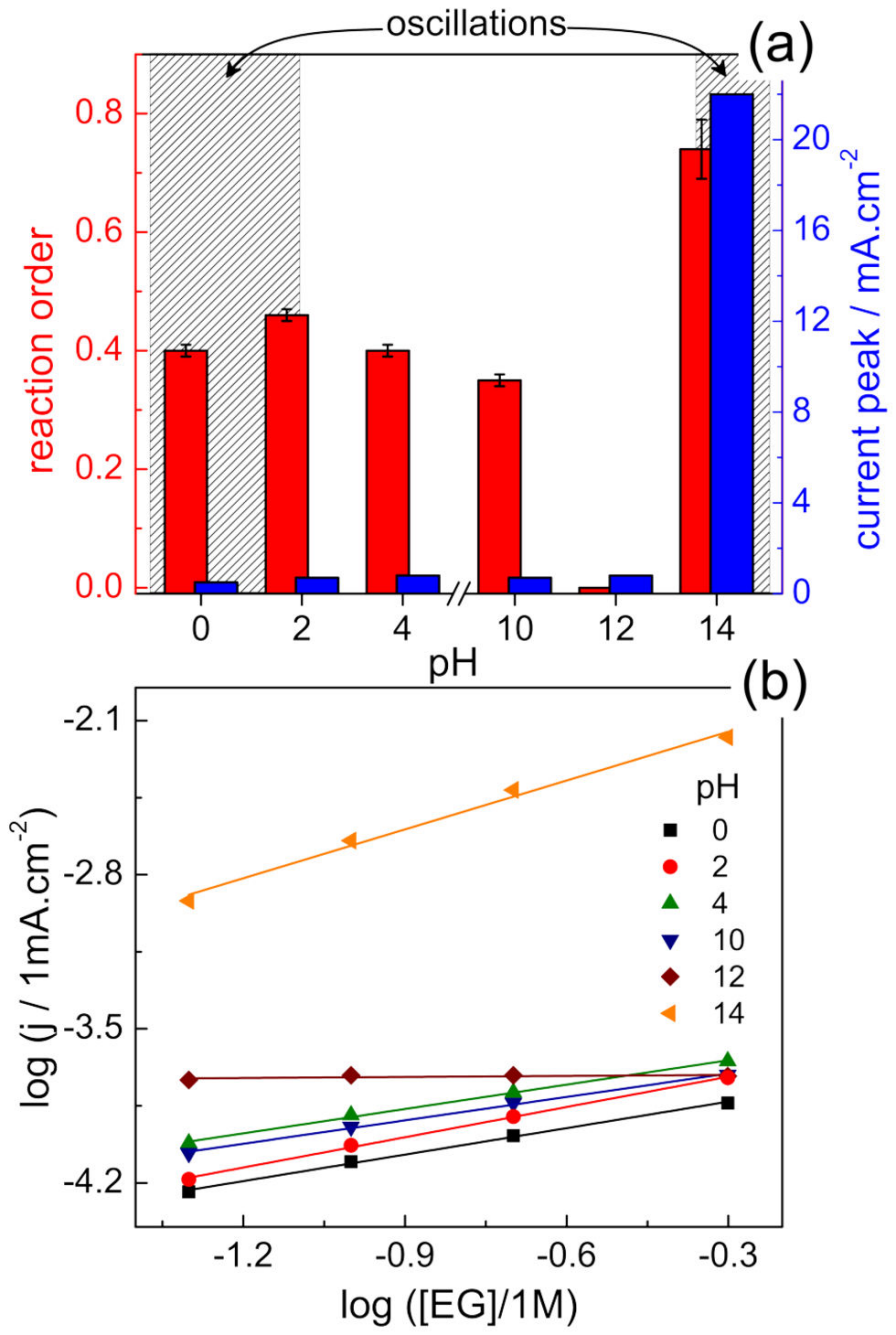
The pH role on EG electro-oxidation reaction. (a) Reaction order (left axis) and current peak (right axis) for several pH values. Reaction order was estimated by current in peak potential over [EG] from 0.05 to 0.5 M. Current peaks refer to [EG] of 0.5 M. (b) log (j) *versus* log [EG] dependence used to estimate reaction orders. Data estimated by the CV currents obtained at 0.01 V.s^-1^ and 20 °C.

Although the rates of reaction are strongly influenced by the EG concentration at most studied pH, the general shape of the current-potential curves remains nearly unaltered. We used plots of log (j) *versus* log [EG] to estimate the reaction order under voltammetric regime at the main current peak. Figure 2(a) shows the obtained reaction orders and the peak current values for each pH, as well as the log (j) *versus* log [EG] curves in plane (b). The estimated reactions orders are about 0.40 ± 0.05 between pH 4 and 10. At pH = 14 the reaction order increases to 0.74 ± 0.05, in line with previously published data [21,36]. Interestingly, at pH = 12 the magnitude of the current peak is hardly influenced by the EG concentration. When log (j) *versus* log [EG] plots are built considering the currents of pre peaks, a non-linear tendency is found at sweep rate of 0.01 V.s^-1^. Fractional reaction orders are usually difficult to rationalize and reflect the role of adsorbed species in the reaction process.

### 2 Oscillations

The switch from potential to current control changes the system’s dynamics as already observed in the red lines in Figure 1. At pH 0 and 2, instabilities in the electrode potentials are observed around the values where the oxidation process starts under potential control. At pH = 14 the instabilities occur in a region close to the current peak. For pH values between 2 and 14 no oscillations are observed.

**Figure 3:**
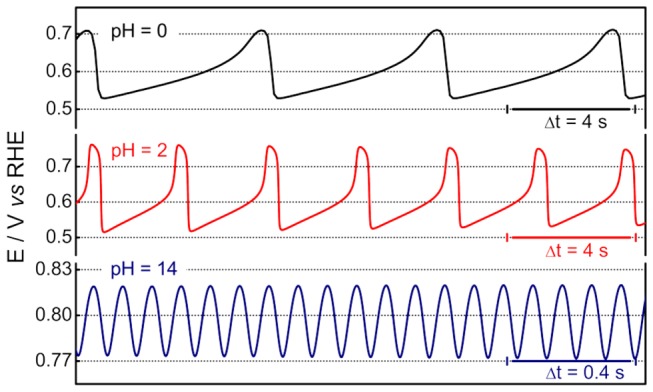
Oscillations patterns registered at pH = 0, 2 and 14. [EG] = 0.5 M. j_N_ = 0.5. See text for details.

Oscillatory electro-oxidation of EG at pH = 1 and around 14 has been previously reported [20,21,25]. In order to compare the oscillatory dynamics under different conditions it is important to maximize the number of parameters that are kept constant in order to rule out influences other than the one is interested in. The applied currents at different pH were normalized in such a way that they would represent equivalent values, accounting for the size of the oscillatory region [8]. Figure 3 shows typical oscillations for a normalized (nondimensional, see ref [8].) current of 0.5, registered near the Hopf bifurcation, i.e. shortly after the birth of potential oscillations. It is noteworthy the different scale used in both axis in bottom panel (pH = 14).

At low pH, relaxation-like oscillations in which the electrode potential slowly increases and then suddenly drops by c.a. 0.25 V, are observed. This pattern has been commonly observed in a vast number of electrochemical oscillators [6,13,22,37] and has been described as a self-organized poisoning/cleaning cyclic process, in which the slowly accumulating poisoning species are oxidized at high potentials. Generally speaking, it can be inferred that the main poisoning species for the oscillations in acidic media is adsorbed carbon monoxide [9,16,38,39,40].

Behm and co-workers [31,41] have suggested CO_ad_ as the poisoning species during the EG electro-oxidation on platinum. They also estimated the rate of CO formation during the adsorption of 0.1 M of EG on platinum and found a maximum near 0.4 V *vs* RHE in a volcano-like curve [41], similar results were found by Fan et al. [42] for a Pt (100) surface. In the light of these works, we assume adsorbed CO is the main poisoning species during de oscillatory EG electro-oxidation on experiments at pH 0 and 2. In fact, the oscillatory patterns in this range remains very similar and basically a decrease in the oscillatory period is observed as the pH is increased.

The patterns change wholly at pH = 14, and oscillations set in as high frequency, low amplitude, quasi-harmonic states. The evolution of such oscillations as well its dependence on the EG concentration has been described in earlier works [20,21,25]. The kinetic of the electro-oxidation of EG is unquestionably faster in alkaline media as seen in the potentiodynamic sweep given in Figure 1. Recalling that oscillations involve the coupling among different individual steps, a less obvious correspondence with the reaction rates under voltammetric regime is in principle expected. Nevertheless, the increase in the oscillation frequency that results from the increase in the solution pH follows the general current increase found under non-oscillatory conditions.

The distribution of the oxidation products of EG in alkaline media is considerably more complex than that in acidic media. A recent systematic study of the electro-oxidation of EG on palladium electrodes [43] showed that intermediate products increase with EG concentration, being glycolate and glyoxalate observed in solutions containing more the 0.1 M of EG. The same trend can be concluded for platinum catalysts, when the references 32] and [33 are compared. The absence of oscillations for EG concentration lower than 0.1 M [21] strongly suggests the role of these intermediates on the oscillatory process. The oxidation products identified by *in situ* FTIRS are glycolate, oxalate and carbonate, on the other hand, the high reactivity of aldehydes makes difficult the detection of this group by conventional methods [44]. Interesting, there is no observable bands of adsorbed CO at potentials where oscillations occur, suggesting that this is not the species responsible for the surface poisoning in alkaline media.

Despite the lack of specific information on the nature of the poisoning species that take part in the oscillatory dynamics in alkaline media, the fact that it is likely that it is not CO_ad_ at potentials that oscillations occurs [34] as in acidic media, seems to be the cause of the main differences in the dynamics at high pH. Some of these aspects will be presented below; at this point the key differences to be highlighted are the oscillation’s waveform, frequency, and amplitude, as well as the potential values visited during the oscillations. The ascending part of the oscillation generally informs on the poisoning process, whereas the descending one reflects the reactivation of the surface towards its high-activity states at low potentials. The sinusoidal character of the patterns at pH = 14 strongly contrasts with the oscillations found in acidic media, which are characterized by a slow poisoning process followed by an abrupt reactivation. In addition, the lower potentials reached during oscillations differ considerably with respect to the electrolyte’s pH and is an additional indication that there is a different poisoning species involved. At pH = 14, this lower limit of more than about 0.77 V is appreciably high when compared to that in acidic media, where CO_ad_ is presumably the poisoning adsorbate. Finally, the high oscillation frequencies found in alkaline media could be though as being in line with the high activity discernible in the voltammetric profile.

**Figure 4:**
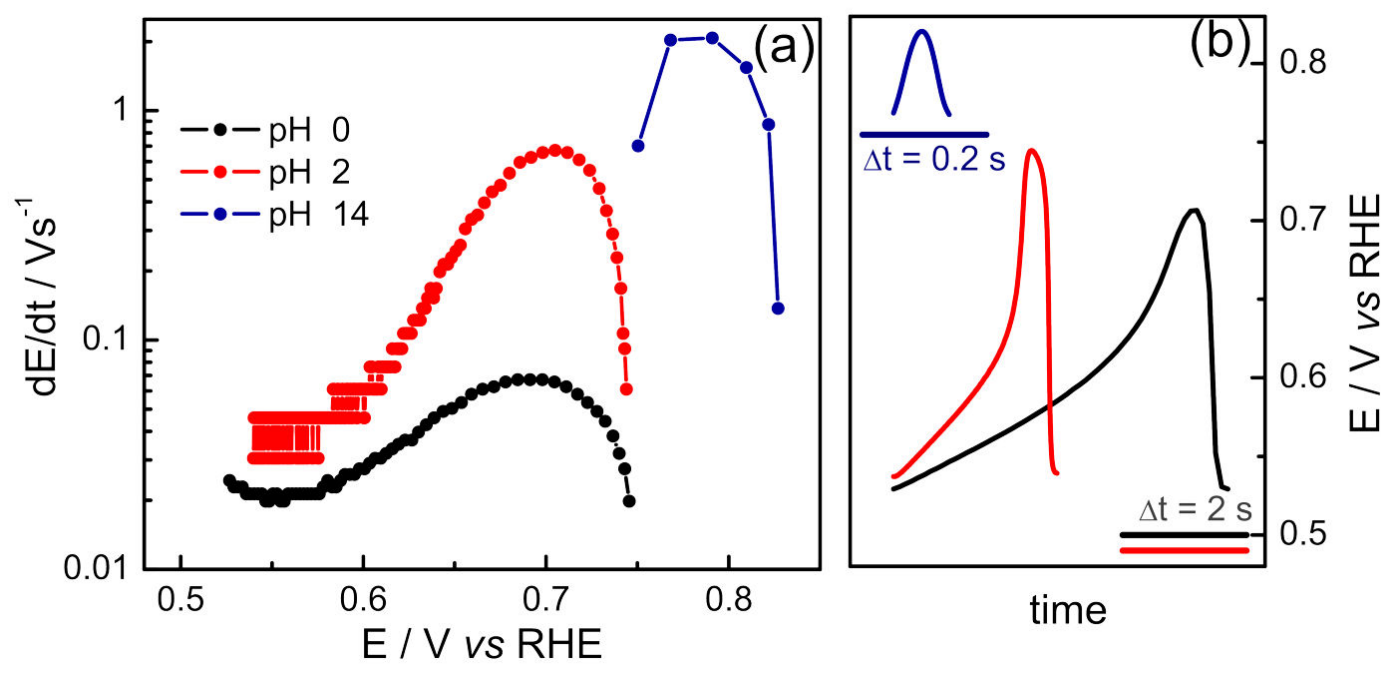
Potential evolution along one oscillation cycle. (a) Potential rate (dE/dt) along the oscillation cycle, and (b) the original cycles presented in (a). The data corresponds to the cycles of Figure 3 for t_N_ = 0.2.

Along the galvanostatic oscillations, the self-organized changes in the electrode potential reveal the complex changes in the population of adsorbates. The increase in the coverage of poisoning species is accompanied by an increase in the electrode potential as the main faradaic reaction is progressively inhibited. In order to accomplish the applied current, the electrode potential increases accordingly up to a point where such adsorbate(s) is(are) oxidized, after that the potential decreases and the whole cycle repeats. Therefore, the spontaneous increase in the electrode potential can be regarded as a measure of the spontaneous poisoning process. This reasoning has been explored in a recent contribution of our Group to the study of the effect of the electrode composition on the surface poisoning [37] and can be employed here to discuss the effect of solution pH. Figure 4(a) brings the evolution of dE/dt as a function of the electrode potential for one oscillatory cycle at distinct pH values, as indicated in panel (b). Clearly, the pH increase shifts dE/dt to higher values, pointing to a faster poisoning process.

Recent works by Kiss et al. [24,45,46] related some oscillatory features with electric and chemical properties and provided important insights on the system’s dynamics. As a typical example, the oscillation frequency has been recently described by the double layer capacitance, the slope of the polarization curve around the bifurcation point, the total resistance, and a rate constant [25]. As a general conclusion, we observed that the higher the adsorption constant of the poisoning species the higher the oscillation frequency and the smaller the amplitude. The same impact on the oscillations’ features is also found for a higher desorption or oxidation constant of the surface blocking species. These theoretical predictions have been confirmed in experiments in rather disparate systems [21,37,47]. In the present case, these predictions shed light on the origin of the faster and smaller amplitude oscillations observed during the electro-oxidation of EG at high pH. In this direction, we can propose that the non-CO_ad_ poisoning species in alkaline media is oxidized faster than that found for CO_ad_ in acidic media. This argument is justified by the higher activity found in alkaline media, as already mentioned, which, in its turn, can be attributed to higher availability of hydroxyl groups to adsorb and oxidize the poisonous species in a Langmuir-Hinshelwood step. Finally, one could not discard the possibility that the poisoning species has a higher adsorption constant than CO at low pH. Nevertheless, even if it were the case, this effect is presumably smaller than the one caused by the higher oxidation rates observed at high pH. Figure 4(a) illustrates the higher rates in alkaline media for both poisoning and subsequent cleaning processes.

**Figure 5:**
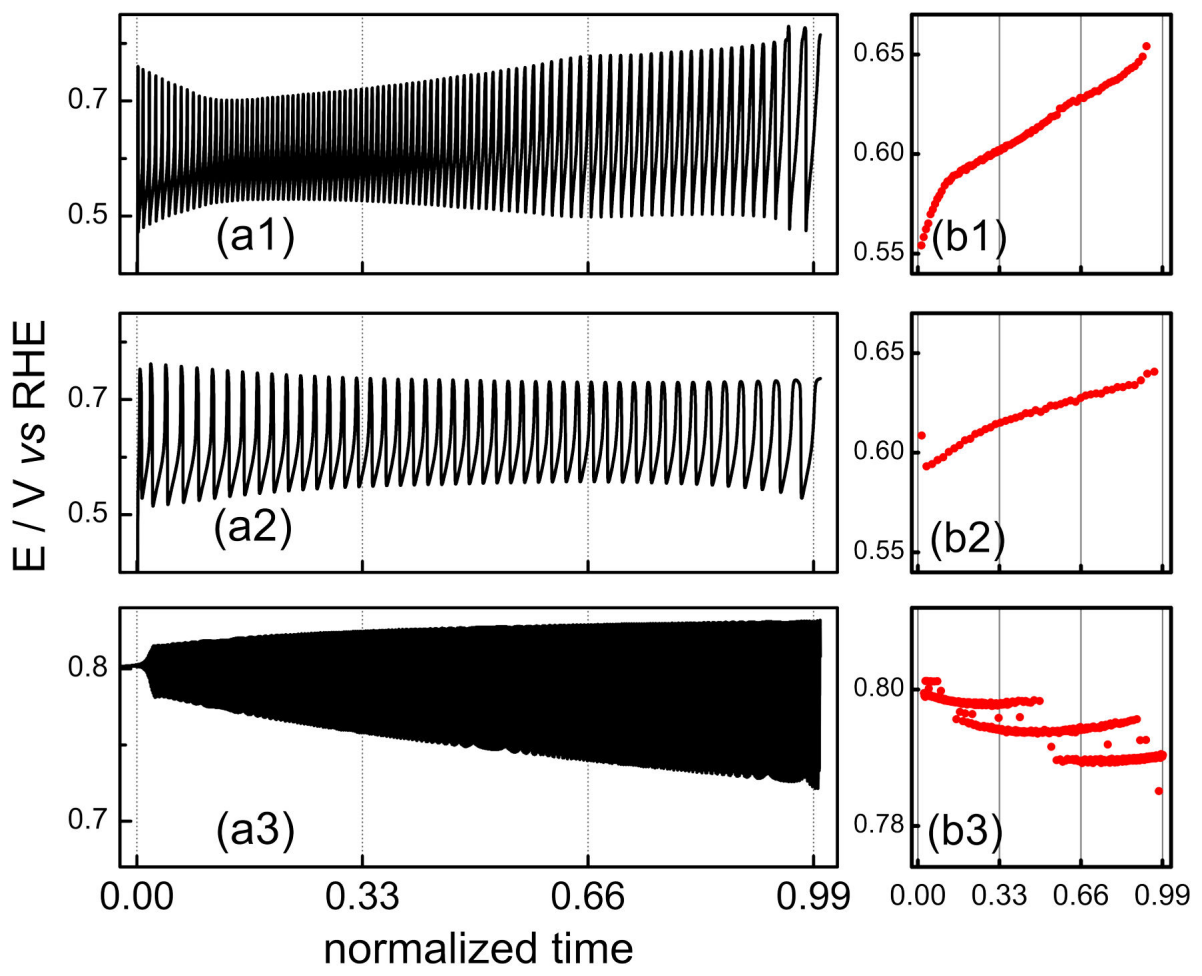
Period-one time-series in terms of normalized time. Red circles represent the mean potential in each cycle. Experimental conditions as in Figure 3.

Concentrating the discussion on the period-one patterns, which are common in all pH where oscillations exist, it is possible to follow the spontaneous changes over time. Figure 5 exemplifies these changes. The variation of the mean electrode potential (E_m_) [13,17] in each cycle is also illustrated in red. These series were normalized in such a way that t = 0 represents the birth of oscillations and t = 1 accounts for the point in which the period-one structure is spontaneously replaced by a different pattern. The analysis of the long-term evolution of the oscillatory dynamics can provide interesting information.

The spontaneous drift has been rationalized as uncompensated oscillations [48], whose main feature consists of an accumulation and/or depletion of adsorbates in a slower timescale than that observed for a single cycle. As a consequence, the spontaneous drift causes a continuous change in the oscillation dynamics, similarly to that if a parameter is deliberately varied. The occurrence of such slow drift can be viewed as equivalent to a process of aging, which informs on the oscillation failure [47]. The spontaneous increase in the mean potential has been also observed in the electro-oxidation of methanol [17,48] and formaldehyde [13]. On the other hand, at pH = 14, a decrease of -18 μV.cycle^-1^ indicates the opposite behavior described above.

The spontaneous drift has been rationalized in terms of the slow formation of platinum oxides [13,48], which occurs concomitantly with the decrease in the coverage of adsorbed carbon monoxide [17]. As the mean electrode potential increases, more CO_ad_ is oxidized by adsorbed oxygenated species. But in the present case, this explanation holds only for results in acidic media. The previously unseen spontaneous decrease of the mean electrode potential at pH = 14, is yet another face of the presence of non-CO poisoning species in alkaline media. As described above, the overall oscillation shape is distinct at high potentials. While at pH 0 or 2 the oscillations are observed immediately after the current is set, at pH 14 there is a considerable induction period [21], and low amplitude cycles are observed near the Hopf bifurcation, in a typical supercritical scenario [49]. The maximum potential reached in each cycle also increases along the series, followed by an increase in the oscillation amplitude.

In the following we investigate the role of the temperature on the electro-oxidation of EG on platinum under different regimes and also at distinct pH values.

### 3 The effect of temperature

**Figure 6:**
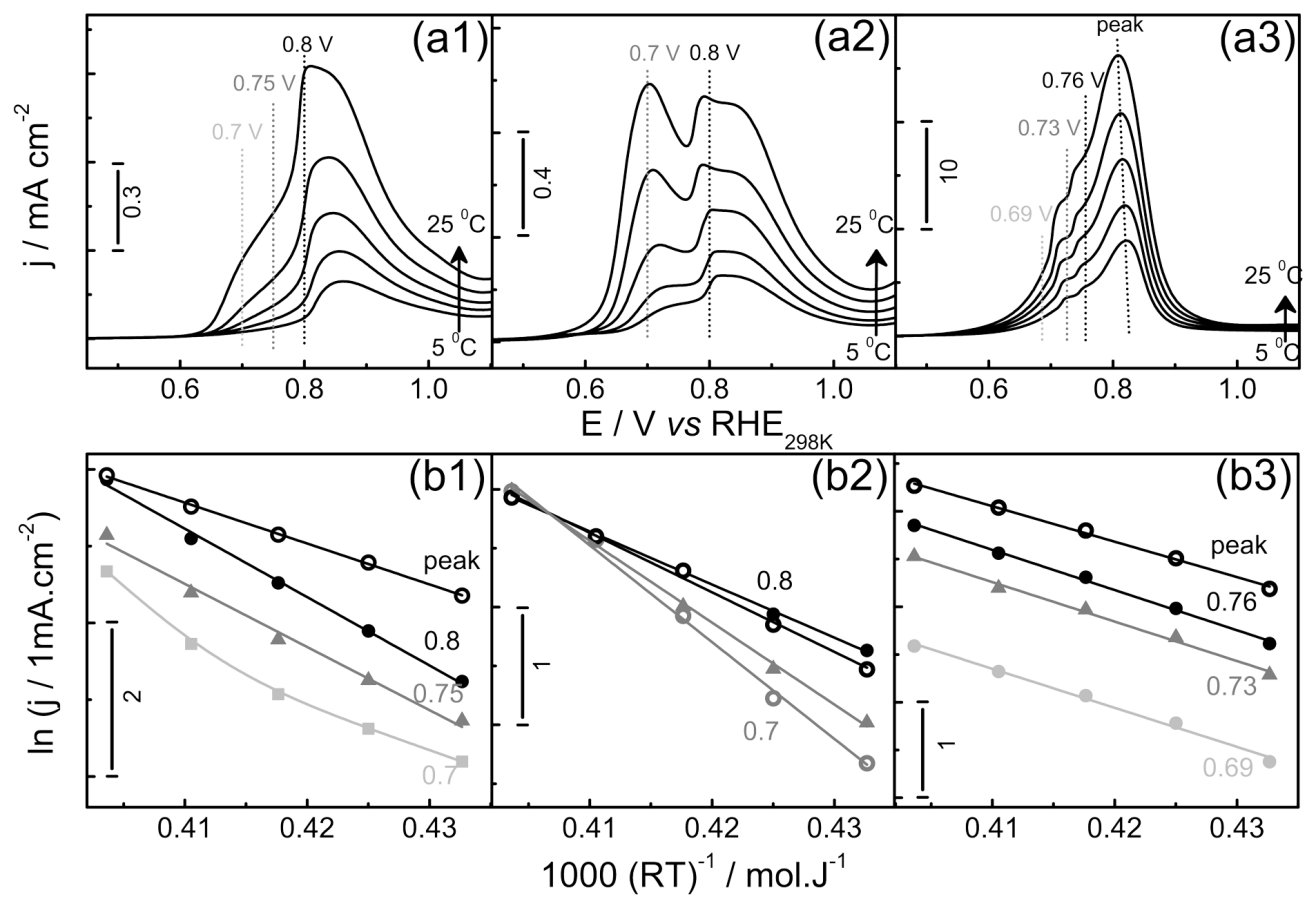
Temperature dependence under potential control. (a) Linear potential sweeps at 0.01 Vs^-1^, at 5, 10, 15, 20 and 25 °C and pH 0 (1), 2 (2) and 14 (3). (b) Arrhenius plot calculated from currents of plane (a). [EG] = 0.5 M.

Figure 6(a) shows linear potential sweeps at 0.01 Vs^-1^ for the EG electro-oxidation in the temperature range from 5 to 25 °C, at pH 0, 2 and 14. The potentials are referred to a reversible hydrogen electrode, corrected by the corresponding value at 298 K [50,51].

The current was found to increase with the temperature in all the cases, while the j-E shapes were maintained. The peak potential is linearly shifted to lower values with temperature increasing, being the highest value found at pH = 0 (-2.4 ± 0.3 mV.K^-1^) followed by pH = 2 (-1.5 ± 0.1 and -1.2 ± 0.2 3 mV.K^-1^ for the first and second peaks, respectively) and pH = 14 (-0.73 ± 0.04 mV.K^-1^). It is important to note that all shifts are negative, in line with the results found for the CO electro-oxidation [52]. The increase of current in systems with high uncompensated resistance shifts the potential to more positive values, probably due to the ohmic drop, leading thus to erroneous interpretations. Herein, despite the low resistance (ca. 3 Ω, estimated by EIS measurements) the curves were corrected to account for the IR term.

**Figure 7:**
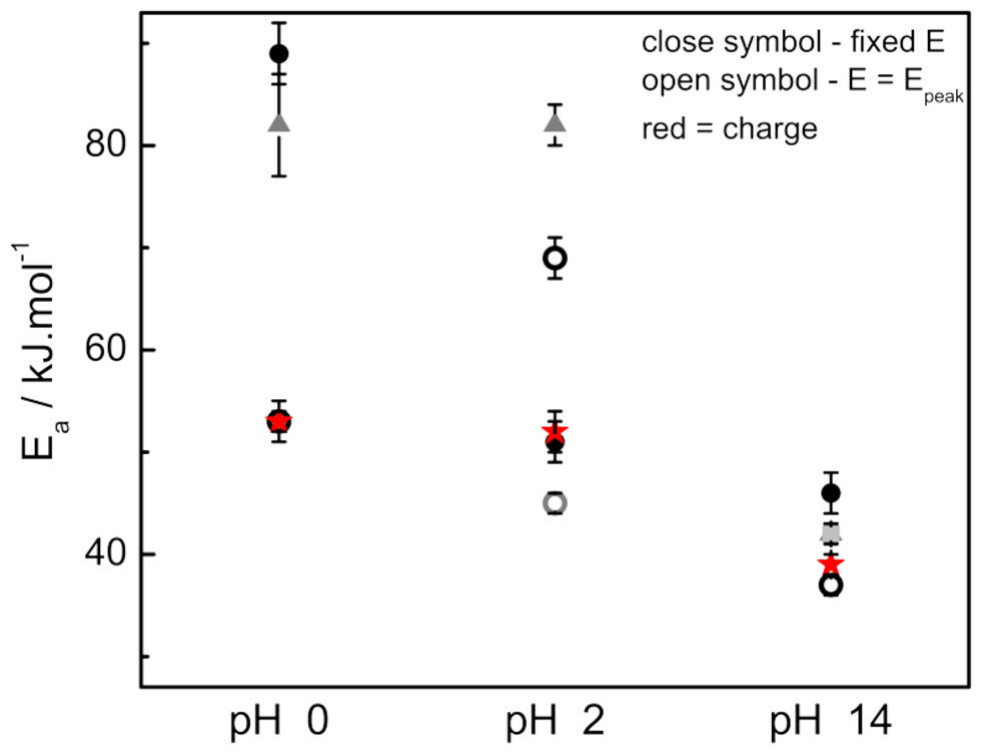
Activation energies estimated under different conditions. The symbol-code is identical to that used in Figure 6. Red stars refer to the E_a_ calculated by means of the charge produced in the positive going scan.

In the planes (b) Arrhenius plots were built considering the current at peak potential (open circles) or fixed potential (closed symbols). At pH=14 the peak is very close to the fixed potential of 0.81 V *vs* RHE. The obtained activations energies (E_a_) are showed in Table 2. The E_a_’ s were also estimated taking into account the charge produced on the sweep window showed in Figure 6(a), and the Arrhenius plots of this parameter are showed in panels (b). The tendency is also showed in Figure 7.

**Table 2:** Activation energies, in kJ.mol^-1^, as estimated by different methods.

	**pH = 0**	**pH = 2**	**pH = 14**
**E_a_ (peak**)	53±1	69±2 (1^st^ peak)	37±1
		45±1 (2^nd^ peak)	
**E_a_ (const. pot.**)	82±5 (0.75 V)	82±2 (0.7 V)	42±1 (0.69 V)
	89±3 (0.8 V)	51±2 (0.8 V)	42±1 (0.73 V)
			46±2 (0.76 V)
**E_a_ (charge**)	53±2	52±2	39±1
**Eω (reg1**)	66±3	72±2	~ 0
**E_ω_ (reg2**)	63±3	74±3	13±2
**E_ω_ (reg3**)	61±3	70±3	18±1

See text for a full description.

At low pH, the activation energy decreases with the electrode potential, and when calculated by the oxidation charge it reaches lower values. The E_a_’ s separation between potential regions decreases with the pH, and at pH = 14 the values corresponds to ca. 40 kJ.mol^-1^ independently of the method or the potential used. These homogeneous energy barriers also permits that the dynamic readily switches to different stages, being a possible explanation for the high oscillations frequencies observed. This feature could also contribute to the more symmetrical shape of oscillations in alkaline than that observed in acid media. In fact, the low oscillatory activation energies at high pH might indicate that the individual activation energies (for elementary steps) are similar and independent on the electrode potential in the relatively narrow window from ~ 0.7-0.8 V.

Independently on the method, all estimated activation energies seem to decrease with the increases of the electrolyte pH. As already discussed, this is likely to be due to the fact that the non-CO_ad_ blocking species at high pH is more easily oxidized than that for CO_ad_ in acidic media.

**Figure 8:**
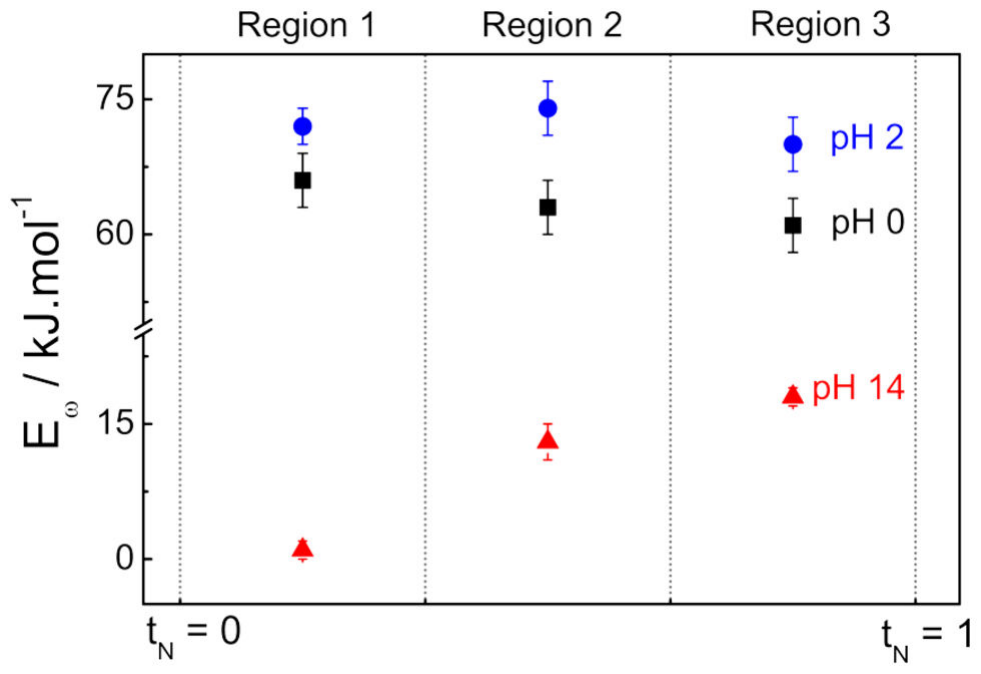
Temporal evolution of the oscillatory activation energy, E_ω_. Data take into account the period-one oscillations at different pH.

The oscillatory activation energy, E_ω_, was estimated with the temperature effect on the oscillation frequency [53] and in terms of the normalized applied current [8,23,26]. As the oscillatory system was found to spontaneously evolve in time. In order to follow the variation of the activation energy as the system spontaneously evolves in time, time-series for the period-1 patterns were normalized in time and divided in three equally spaced regions. The E_ω_ were estimated in each case using the mean oscillatory frequency. Figure 8 shows the E_ω_ along the spontaneous evolution of the time-series.

Region 1 encompasses the initial instants after oscillations set in. As discussed in an early publication [20,21], in alkaline media, oscillations are almost insensitive to temperature changes, leading to E_ω_ around zero, in the so-called temperature compensation [8,54,55]. At low pH, this phenomenon was not observed, as proved by E_ω_ values on the same range observed in that ones calculated under voltammetric conditions. As the system evolves, at low pH, E_ω_ remains nearly constant, in agreement with the slightly varying morphology of the potential oscillations. On the other hand, at pH = 14 the tendency is the opposite, and E_ω_ increases from near zero to about 18 kJ.mol^-1^. The Region 3, comprehends the extinction of the period-one oscillations, process that extinguish the oscillations at pH = 0 or changes the oscillation morphology at pH 2 and 14.

The acceleration of both processes of surface poisoning and cleaning under oscillatory regime at pH = 14, c.f. Figure 4(a), might well be the cause of the tendency of finding temperature compensation. The opposite trend observed in the evolution of E_ω_ is yet another aspect of the different poisoning species observed in acidic and in alkaline media. Table 2 summarizes all relevant activation energies estimated here.

## Summary and Concluding Remarks

The comprehensive study of the electro-oxidation of ethylene glycol (EG) reported here covers the investigation of the effects exerted by the electrolyte pH, the EG concentration, and temperature, under both regular and oscillatory conditions. The key findings can be summarized as follows:

a Despite the additional complications evidenced in the multiple-wave structures of the potentiodynamic sweep, the current remains comparatively small at pH from 0 to 12 and increases about 25 times at pH 14;
b The reaction order with respect to EG concentration, estimated under voltammetric regime, was found to increase from a constant value of c.a. 0.4-0.5 (pH from 0 to 10) to 0.8 at pH = 14. Remarkably, reaction orders around zero were found at pH = 12;
c Potential oscillations were found at low (0 and 2) and high (14) pH. Oscillations are considerably faster at pH = 14 and the general waveforms are considerably different in acidic and in alkaline media;
d At high pH, the mean oscillating potentials are higher than that in acidic media and the rate of poisoning is more than 10 times faster with respect to that in pH = 0;
e The long term evolution of the galvanostatic oscillations evidences that the mean electrode potential generally increases in acidic media but decreases at pH = 14;
f The effect of temperature was studied from 5 to 25 °C and the apparent activation energy estimated under different conditions. Under voltammetric regime, the activation energy as estimated by the total oxidation charge, it remains about 50 kJ.mol^-1^ in acidic media and drops to 40 kJ.mol^-1^ at pH = 14. Furthermore, in acidic media E_a_ decreases with the electrode potential, whereas at pH = 14, it is hardly influenced by the electrode potential and the estimation method;
g Under oscillatory regime, E_ω_ values are about 66-72 kJ.mol^-1^ at pH = 0 and 2, and near zero at pH = 14. E_ω_ decreases (increases) due to the spontaneous drift in acidic (alkaline) media.

As the main lesson, we found that increasing the electrolyte pH results in a discontinuous transition in most kinetic parameters and properties investigated under both voltammetric and oscillatory regimes. As a relevant result in this direction is the increase in the reaction order with respect to the concentration of ethylene glycol with pH from 0.4-0.5 to 0.8, which is mediated by a minimum (~ 0) at pH = 12. The distinction between the kinetics at low and high pH is in line with some mechanistic findings from earlier reports. Previous studies indicate that CO_ad_ plays a central role as poisoning species at relatively low potentials, and glycolaldehyde and glycolic acid as the main products. In alkaline media, the electro-oxidation of EG on platinum results in carbonate, oxalate, glycolate and glyoxylate, and there is no evidence of appreciably amount of adsorbed CO.

Following these mechanistic considerations, it seems undisputable that the mechanisms underlying the oscillatory dynamics at low and high pH values are intrinsically different. The absence of oscillations at intermediate pH corroborates this hypothesis. As already mentioned, the surface-blocking species is probably CO_ad_ in acidic media and other species in alkaline media. One can speculate that the adsorption isotherms of different species, c.f. carbonaceous and oxygenated species, do not overlap properly to allow the emergence of potential oscillations.

Altogether, there is apparently a correspondence between the two sets of data under regular and oscillatory regimes, as for instance the high voltammetric activity and the high oscillation frequency registered at pH = 14. Both the amount of comparable parameters and properties presented in this study and the discussion that followed illustrate the strategy of combining studies under regular and oscillatory conditions for the investigation of electrocatalytic systems.
